# Multimodal Computed Tomography Increases the Detection of Posterior Fossa Strokes Compared to Brain Non-contrast Computed Tomography

**DOI:** 10.3389/fneur.2020.588064

**Published:** 2020-11-20

**Authors:** Cecilia Ostman, Carlos Garcia-Esperon, Thomas Lillicrap, Shinya Tomari, Elizabeth Holliday, Christopher Levi, Andrew Bivard, Mark W. Parsons, Neil J. Spratt

**Affiliations:** ^1^Department of Neurology, John Hunter Hospital, Newcastle, NSW, Australia; ^2^Hunter Medical Research Institute and University of Newcastle, Newcastle, NSW, Australia; ^3^School of Medicine and Public Health, University of Newcastle, Newcastle, NSW, Australia; ^4^Maridulu Budyari Gumal, The Sydney Partnership for Health Education Research & Enterprise (SPHERE), University of New South Wales, Sydney, NSW, Australia; ^5^Melbourne Brain Centre at the Royal Melbourne Hospital, University of Melbourne, Parkville, NSW, Australia; ^6^UNSW South Western Sydney Clinical School, University of New South Wales and Department of Neurology, Liverpool Hospital, Liverpool, NSW, Australia

**Keywords:** CT perfusion, multimodal CT, posterior fossa stroke, mean transit time, delay time

## Abstract

**Aims:** Multimodal computed tomography (mCT) (non-contrast CT, CT angiography, and CT perfusion) is not routinely used to assess posterior fossa strokes. We described the area under the curve (AUC) of brain NCCT, WB-CTP automated core-penumbra maps and comprehensive CTP analysis (automated core-penumbra maps and all perfusion maps) for posterior fossa strokes.

**Methods:** We included consecutive patients with signs and symptoms of posterior fossa stroke who underwent acute mCT and follow up magnetic resonance diffusion weighted imaging (DWI). Multimodal CT images were reviewed blindly and independently by two stroke neurologists and area under the receiver operating characteristic curve (AUC) was used to compare imaging modalities.

**Results:** From January 2014 to December 2019, 83 patients presented with symptoms suggestive of posterior fossa strokes and had complete imaging suitable for inclusion (49 posterior fossa strokes and 34 DWI negative patients). For posterior fossa strokes, comprehensive CTP analysis had an AUC of 0.68 vs. 0.62 for automated core-penumbra maps and 0.55 for NCCT. For cerebellar lesions >5 mL, the AUC was 0.87, 0.81, and 0.66, respectively.

**Conclusion:** Comprehensive CTP analysis increases the detection of posterior fossa lesions compared to NCCT and should be implemented as part of the routine imaging assessment in posterior fossa strokes.

## Introduction

The posterior fossa is the area located at the base of the skull which contains the brainstem and the cerebellum, and this region accounts for 13% of all ischemic strokes (1, 2). Posterior fossa strokes are often misdiagnosed, as some patients present acutely with non-specific symptoms such as vertigo (47%), dysarthria (31%), or nausea (27%) (3). As a result, these patients have longer times to reperfusion therapies compared to anterior circulation strokes (4). Delays in acute diagnosis may also lead to delays in identifying life-threatening complications of ischemic strokes in the posterior fossa such as occlusive hydrocephalus or brainstem compression secondary to ischemia (5). These complications occur in up to 40% of cerebellar strokes, in particular those with large volumes of ischemic tissue (larger than 5 mL) (2, 5).

The use of multimodal computed tomography (mCT), defined as the combination of non-contrast CT (NCCT), CT angiography (CTA), and CT perfusion (CTP) to identify candidates for acute stroke reperfusion therapies is now common practice for anterior ischemic strokes in the 24-h window (6, 7). However, its use in the posterior fossa for clinical-decision making has not been as widely accepted. This is mostly due to physiological factors, such as the beam hardening artefact created by bony interference of the cranial vault, and technical factors such as limited z-axis coverage and high variability between automated perfusion software and CT vendor software (8–10).

With the advent of new technology, many current CT machines are able to perform whole brain CTP (WB-CTP) (11). Moreover, automated perfusion software is now widely available and commonly used (6, 7, 12). Perfusion deficits on WB-CTP have previously been shown to predict final infarct volume in the cerebellum, and the development of malignant cerebellar oedema (13, 14). However, whether WB-CTP can detect lesions in the posterior fossa in an undifferentiated patient population who present with symptoms of posterior fossa stroke remains unknown.

We aimed to (a) describe the sensitivity, specificity, negative and positive predictive values and area under the receiver operating characteristic curve (AUC) of WB-CTP in a group of consecutive patients presenting with symptoms suggestive of posterior fossa stroke and (b) to determine the influence of lesion volume on sensitivity, specificity, negative and positive predictive values and AUC of WB-CTP in posterior fossa strokes. Our principal hypothesis was that WB-CTP would increase the accuracy of detecting posterior fossa strokes compared to brain NCCT, and in particular, would increase the detection of cerebellar strokes >5 mL.

## Materials and Methods

### Population and Data Collection

Retrospective analysis of consecutively recruited suspected strokes presenting to the John Hunter Hospital (New South Wales, Australia) from January 2014 to December 2019 who underwent acute mCT. Patients were included if they presented with signs and symptoms compatible with posterior fossa stroke (defined as a combination of dysarthria, unsteadiness, headache, limb weakness, drowsiness, diplopia, nausea, nystagmus, or dizziness), and underwent multimodal CT imaging in the acute setting (within 24 h from symptom onset) and follow up diffusion weighted (DWI) magnetic resonance imaging (MRI) within 7 days. Stroke patients were defined as those with a confirmed infarct in the brainstem or cerebellum on DWI and DWI negative patients as those without DWI lesion but who presented with signs and symptoms compatible with posterior fossa stroke. Data collected included baseline demographics, stroke severity—measured by the National Institutes of Health Stroke Scale (NIHSS) on presentation -, and baseline and follow up image characteristics on mCT (presence and location of infarct) and DWI (presence, location, and volume of infarct).

### Imaging Protocol

The imaging protocol included baseline mCT and follow-up DWI as per clinical routine. WB-CTP was performed using a 320-Slice Toshiba Aquilion One scanner (Canon Medical Systems, Otawara, Japan), with a z-axis of 150 mm. To acquire WB-CTP a 50 mL bolus of iodinated contrast agent at a rate of 6 mL/s was injected through cubital fossa venous access, with a total acquisition time of 72 s. A 1.5 or 3 Tesla MRI scanner was used (Siemens Aera/Siemens Verio, Siemens AG, Healthcare Sector, Erlangen, Germany). All CTP imaging was post-processed using the commercial software MIStar (Apollo Medical Imaging Technology, Melbourne, Australia) to generate automated core-penumbra maps as well as cerebral blood volume (CBV), cerebral blood flow (CBF), mean transit time (MTT) and delay time (DT) maps. Penumbra was defined as the tissue with a DT≥3 s and relative CBF≥30% of the contralateral hemisphere. Ischemic core was defined as the tissue with a DT≥3 s and a relative CBF <30% of the contralateral hemisphere (15). All images were reprocessed and analysed using the same version of the MIStar software (Version 3.2 release 3.2.62.03, last update October 2019).

### Image Assessment

All mCT images were reviewed independently by two neurologists who were blinded to the final diagnosis, but unblinded to the initial clinical presentation (including side) and NIHSS. CTP maps assessed included automated core-penumbra maps and all post-processed maps including Cerebral Blood Volume (CBV), Cerebral Blood flow (CBF), Mean Transit Time (MTT), and Delay Time (DT). The neurologists were required to assess the presence or absence of ischemic changes in the posterior fossa (right cerebellum, left cerebellum and/or brainstem) in any of the mentioned maps and to classify the patient as posterior fossa stroke or not. All DWI-MRI images were reviewed by a third reviewer (blinded to the results of the mCT) who was required to assess the presence or absence of ischemic changes in the posterior fossa (right cerebellum, left cerebellum and/or brainstem). The neurologists assessing mCT images remained blinded to the MRI images.

### Statistical Analysis

All analyses were completed using the statistical software Stata version 15 (StataCorp LLC, Texas, United States). Groups were compared using Chi square tests, independent sample *t*-tests or Mann–Whitney *U*-tests as appropriate. Sensitivity, specificity, negative predictive value, positive predictive value, and area under the receiver operating characteristic curve (AUC) were calculated based on CTP lesion detection compared to the follow up DWI. A sub-analysis of cerebellar lesions was performed based upon total lesion volume (as assessed by DWI) to determine the influence of lesion volume on detection by mCT. Unless otherwise mentioned, median values are followed by [interquartile range], and mean values are followed by (standard deviation). Statistical estimates (such as AUC) are followed by (95% confidence interval). Results presented here are the mean for the combined raters, with data regarding individual raters and inter-rater variability presented in the Supplementary Material. To calculate these averages, each patient was treated as 2 separate data-points for each assessment (1 per rater) and analyses were performed across the resulting assessments.

### Ethics Approval

The study had approval from the Hunter New England Local Health District Human Research Ethics Committee in accordance with Australian National health and Medical Research Council guidelines (Reference No: 11/08/17/4.01).

## Results

From January 2014 to December 2019, 833 consecutive patients who presented with stroke-like symptoms and underwent acute mCT and follow up MRI were analysed. On follow-up MRI, 60 patients had a confirmed diagnosis of posterior fossa stroke, and 43 patients were not strokes but presented with symptoms of posterior fossa stroke (DWI negative patients). Of these 103 patients, a total of 20 patients were excluded (19 because of lack of access to all CTP maps and one patient because of movement artefact). Therefore, 83 patients were included in this analysis–including 49 posterior fossa stroke patients and 34 DWI negative patients.

The median age of the confirmed strokes was 71 years [59–78], compared to 59 [47–66] years old in the DWI negative group (*p* = 0.001), and the median baseline NIHSS score was 4 in the stroke group vs. 3 in the DWI negative group (*p* = 0.32) (Table 1). Equal numbers of the stroke patients had an isolated cerebellar lesion and single brainstem lesions (20 cases, 41% in each group), and 9 (18%) had lesions in both the cerebellum and brainstem (Table 1). Sixteen posterior fossa patients had co-existing lesions in the occipital lobe or thalamus. The median overall DWI lesion volume among the stroke patients was 0.51 [0.2–2.7] mL. The median cerebellar lesion volume was 1.24 [0.2–10] mL and the median brainstem volume was 0.33 [0.2–0.6] mL. Eleven stroke patients had lesions larger than 5 mL, and these were all located in the cerebellum, with a median lesion volume of 17.2 [7.9–19.9].

**Table 1:** Patient characteristics of confirmed strokes and DWI negative patients.

	**Posterior fossa strokes (*n* = 49)**	**DWI negative patients (*n* = 34)**	***p*-value**
Median age–year [IQR]	71 (59–78)	59 [47–66]	0.001
Median NIHSS score [IQR]	4 [3–9]	3 [1–7]	0.90
Female sex–no (%)	18 (37%)	15 (44%)	0.50
Clinical history–no (%) Hypertension Diabetes mellitus Atrial fibrillation Dyslipidaemia	35 (71%) 7 (14%) 11 (22%) 21 (43%)	14 (41%) 3 (8%) 4 (11%) 11 (32%)	<0.01 0.45 0.21 0.33
Stroke location–no (%)			
Cerebellum	20 (41%)		
Brainstem	20 (41%)		
Both	9 (18%)		
Stroke treatment–no (%) Thrombolysis Endovascular thrombectomy Combined therapy	5 (10%) 3 (6%) 7 (14%)		
DWI negative patients' diagnosis–no (%) Migraine Undetermined Functional neurological disorder Exacerbation of underlying neurological symptoms Headache syndromes other than migraine Benign paroxysmal positional vertigo Syncope Seizure Other: Infection, acute inflammatory demyelinating polyneuropathy, vestibular neuritis, hyperglycaemia, delirium		7 (21%) 7 (21%) 3 (9%) 3 (9%) 3 (9%) 2 (6%) 2 (6%) 2 (6%) 5 (15%)	

*NIHSS, National Institutes of Health Stroke Scale*.

### Brain NCCT vs. CTP

**Figure 1:**
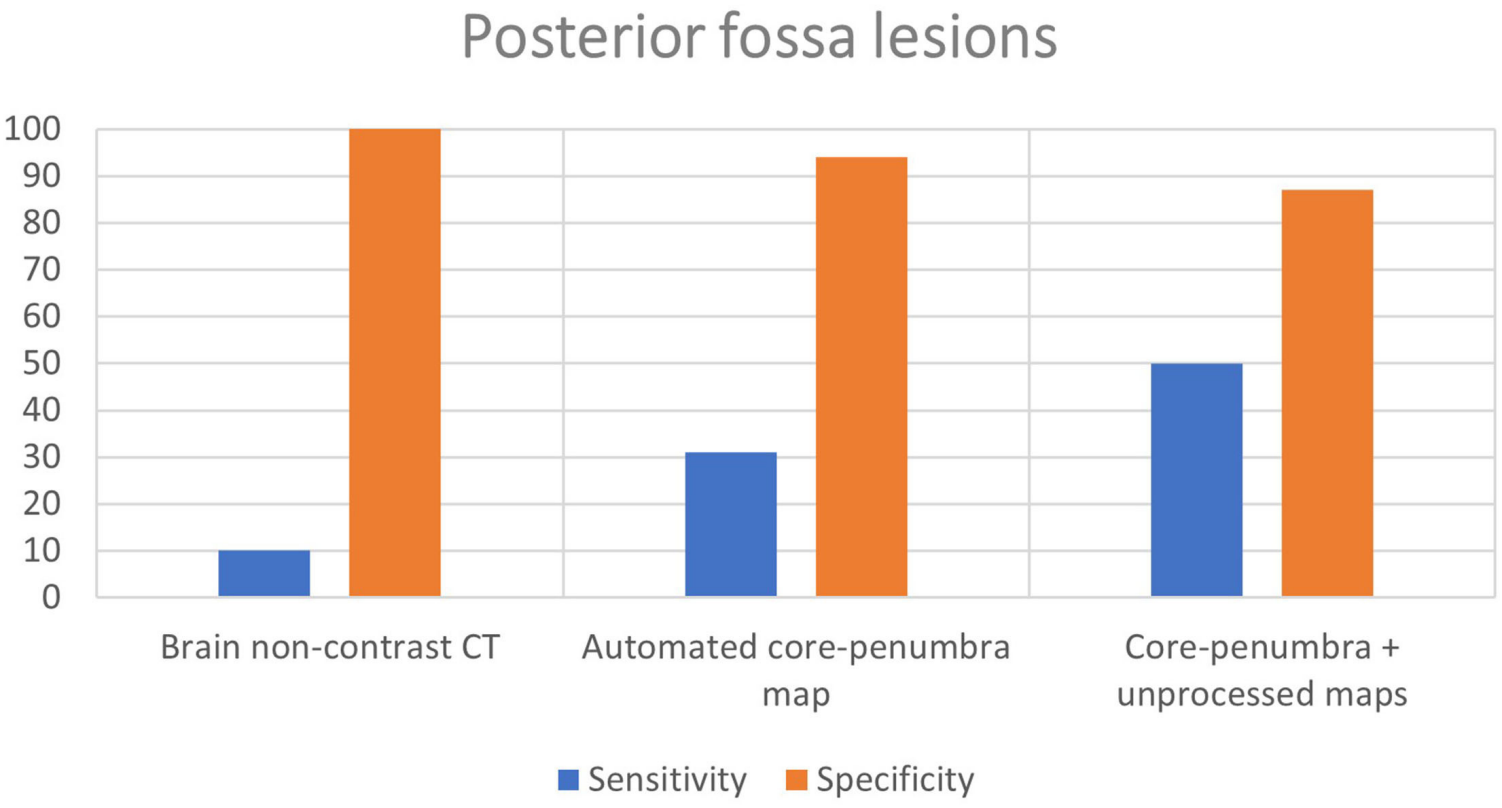
Mean sensitivity and specificity for detection of posterior fossa lesions of different imaging modalities.

Brain NCCT had poor sensitivity (10%) but high specificity (100%) for posterior fossa strokes. The mean sensitivity of the two assessors using the CTP automated core-penumbra maps was 31%, which increased further to 50% for comprehensive CTP analysis (automated core-penumbra map + all perfusion maps). Both the automated core-penumbra map and comprehensive CTP analysis had good specificity in the posterior fossa (94 and 87%, respectively). These findings are demonstrated in Figure 1 and the data for individual raters is included in the supplementary material (Supplementary Table 1).

**Figure 2:**
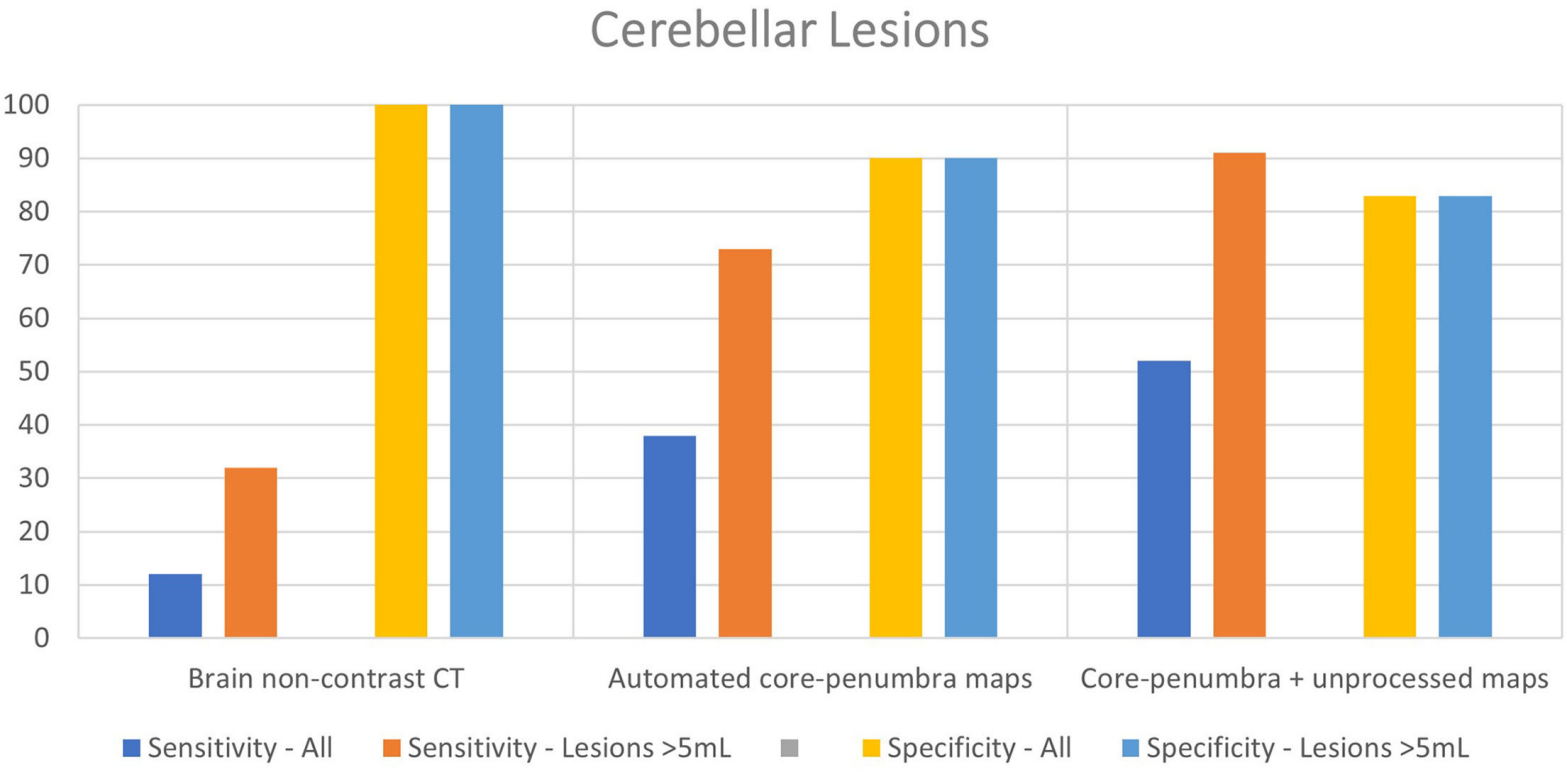
Sensitivity and specificity of different imaging modalities for cerebellar lesions overall and cerebellar lesions>5 mL.

**Figure 3:**
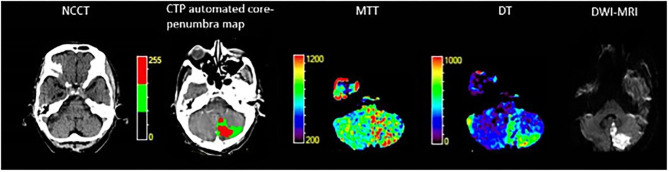
74-year-old male who presented with vertigo, left dysmetria and left sided weakness. NCCT, Non contrast computed tomography; CTP, Computed tomography perfusion; MTT, Mean transit time; DT, Delay time; DWI-MRI, Diffusion weighted imaging—magnetic resonance imaging.

Twenty-nine of the included stroke patients had cerebellar lesions (20 with isolated cerebellar lesion, 9 with combination of cerebellar and brainstem lesion). Brain NCCT had a poor sensitivity (12%) with high specificity in the cerebellum (100%). The CTP automated core-penumbra map had a mean sensitivity of 38%, and comprehensive CTP analysis had a mean sensitivity of 52%, both with good specificity (90 and 83%, respectively). In the subgroup of patients with a cerebellar lesion larger than 5 mL (*n* = 11), the sensitivity of NCCT was 32%, increasing to 73% for CTP automated core-penumbra maps and to 91% for comprehensive CTP analysis. The specificities for the same modalities were 100, 90, and 83%, respectively. These results are displayed in Table 2 and Figure 2. An example of a patient with a cerebellar lesion is demonstrated in Figure 3.

**Table 2:** Sensitivity and specificity for cerebellar lesions with different imaging modalities.

	**All cerebellar lesions**			**Cerebellar lesions** **>5 mL**		
	**NCCT**	**Automated core-penumbra map**	**Automated core-penumbra map + unprocessed maps**	**NCCT**	**Automated core-penumbra map**	**Automated core-penumbra map + unprocessed maps**
True positive	7	22	30	7	16	20
False positive	0	11	18	0	11	18
False negative	51	36	28	15	6	2
True negative	108	97	90	108	97	90
Sensitivity	12%	38%	52%	32%	73%	91%
Specificity	100%	90%	83%	100%	90%	83%

### Area Under the Receiver Operating Characteristic Curve of Brain NCCT vs. CTP

In the posterior fossa overall, brain NCCT had a very poor AUC of 0.55 [0.52–0.58]. Automated core-penumbra maps and comprehensive CTP analysis had relatively poor AUC of 0.62 [0.57–0.68] and 0.68 [0.62–0.75], respectively. However, the CTP automated core penumbra map and comprehensive CTP analysis demonstrated a good AUC for cerebellar lesions >5 mL [0.81 (0.71–0.91) and 0.87 (0.80–0.94), respectively] compared to poorer AUC for NCCT [0.66 (0.56–0.76)] (Table 3; Supplementary Table 2). The receiver operating characteristic curves are included in the supplementary materials (Supplementary Figures 1–3). A *post-hoc* analysis was conducted where patients who received reperfusion therapy were excluded from the analysis. In the posterior fossa, the AUC of comprehensive CTP analysis was 0.68 (0.60–0.75). The AUC was 0.88 (0.79–0.97) in patients with cerebellar lesions>5 ml. These results are included in the supplementary materials (Supplementary Table 3).

**Table 3:** Area under the curve (with 95% confidence intervals) for the detection of posterior fossa strokes for different imaging modalities.

	**Posterior fossa strokes**	**Cerebellar strokes**	**Cerebellar strokes >5 mL**
Brain non-contrast CT	0.55 (0.52–0.58)	0.56 (0.52-0.60)	0.66 (0.56-0.76)
Automated core-penumbra map	0.62 (0.57–0.68)	0.64 (0.57-0.71)	0.81 (0.71-0.91)
Automated core-penumbra + unprocessed maps	0.68 (0.62–0.75)	0.68 (0.60-0.75)	0.87 (0.80-0.94)

## Discussion

We have shown that a comprehensive CTP analysis including all perfusion maps (CBV, CBF, MTT, and DT) improves the detection of posterior fossa strokes, compared to brain NCCT and CTP automated core-penumbra maps alone. This analysis is particularly good for detecting cerebellar lesions that are larger than 5 mL. These findings are particularly relevant, since examination findings tend to be less specific for cerebellar lesions than for anterior circulation strokes (3, 16). Previous studies of WB-CTP in posterior circulation strokes have reported sensitivity of 76.6%, specificity of 92.4%, and AUC of 0.86 (17, 18), but these studies relied on NCCT as standard follow-up imaging, with gold-standard MRI only performed on a sub-set of patients, and no specific analysis with regard to lesion size has previously been performed.

Our findings show that the comprehensive analysis of CTP is quite accurate to detect those cerebellar strokes which are large enough to develop clinically significant oedema. This may be useful to identify those patients who are at risk of further neurological deterioration, especially those who are distant to a comprehensive stroke centre with neurosurgical access. Multimodal CT is increasingly available in Australia and other countries, and is used in rural hospitals that are supported by telestroke networks (19, 20). Our results provide yet another reason to support use of mCT in rural and remote areas.

A comprehensive assessment of all CTP maps is complex, and it requires experience in the interpretation (as opposed to simply relying on the automated core/penumbra map output). In our study, the assessors had different levels of experience with CTP interpretation (3 years vs. >5 years of experience). As a result, the sensitivity of a comprehensive CTP analysis ranged from 39 to 61% for lesions in the posterior fossa. These findings highlight further the need to train stroke neurologists in the assessment of all CTP maps (as well as NCCT) to increase the sensitivity of the CTP for detection of posterior fossa lesions (21, 22).

DWI-MRI is the gold standard for detection of acute ischemic lesions. The accuracy of mCT is still limited for small ischemic lesions, especially those in the posterior fossa. The potential implication of our findings is that stroke mimics could be misclassified as ischemic strokes, and potentially offered thrombolysis. Reassuringly, the risk of intracerebral haemorrhage with thrombolysis in stroke mimics is reported to be low (0.5%) (23). The higher sensitivity of comprehensive CTP analysis found in our study can increase the yield of posterior fossa stroke identification in the acute setting.

There are several limitations to this study. Firstly, the number of patients with confirmed posterior fossa lesions is relatively small. In particular, only 11 patients had cerebellar lesions larger than 5 mL. In terms of CTP analysis, it is possible that TIAs were classified as DWI negative in those patients whose symptoms resolved prior to DWI-MRI. Furthermore, patients who had simultaneous thalamic or occipital lesions were not excluded from the analysis and this may affect the adjudication of the raters. Moreover, some patients received reperfusion therapies, which may affect the final volumes found on MRI compared to the initial CTP lesions. However, when patients who received reperfusion therapies were excluded from the analysis, the AUC of comprehensive CTP analysis did not change significantly.

In conclusion, our findings show that CTP analysis, especially in combination with review of all perfusion maps, increases identification of posterior fossa strokes compared to brain NCCT alone. Our results suggest that stroke physicians (and other stroke speciality groups) should receive specific training in acute CTP map interpretation.

## Data Availability Statement

The raw data supporting the conclusions of this article will be made available by the authors, without undue reservation.

## Ethics Statement

The studies involving human participants were reviewed and approved by Hunter New England Local Health District Human Research Ethics Committee, Hunter New England Local Health District. Written informed consent for participation was not required for this study in accordance with the national legislation and the institutional requirements. Written informed consent was not obtained from the individual(s) for the publication of any potentially identifiable images or data included in this article.

## Author Contributions

CO and CG-E contributed equally to all aspects of the project and share first authorship. TL and EH carried out statistical analysis. ST was involved in image analysis. CL, AB, and MP were involved in research design and evaluation. NS supervised the project and was involved in design and critical evaluation. All authors were involved in manuscript preparation.

## Conflict of Interest

The authors declare that the research was conducted in the absence of any commercial or financial relationships that could be construed as a potential conflict of interest.
