# Supplementary material for: Multimodal Computed Tomography Increases the Detection of Posterior Fossa Strokes Compared to Brain Non-contrast Computed Tomography
Source: Front Neurol. 2020 Nov 20;11:588064. doi: 10.3389/fneur.2020.588064 (PMC7714905; doi:10.3389/fneur.2020.588064)

Supplementary Materials:

*Table 1.* Sensitivity, specificity, positive predictive value, negative predictive value, and inter-rater agreement (Kappa) across three imaging modalities for posterior fossa strokes.

|  |  | Sensitivity | Specificity | PPV | NPV | Inter-rater agreement (Kappa) |
| --- | --- | --- | --- | --- | --- | --- |
| NCCT | Rater 1 | 12% | 100% | 100% | 44% |  |
|  | Rater 2 | 8% | 100% | 100% | 43% | 0.58 |
| Automated core-penumbra map | Rater 1 | 29% | 97% | 93% | 49% |  |
|  | Rater 2 | 33% | 91% | 84% | 48% | 0.71 |
| Automated core-penumbra + unprocessed maps | Rater 1 | 61% | 82% | 83% | 60% |  |
|  | Rater 2 | 39% | 91% | 86% | 51% | 0.49 |

*Table 2*. The area under the receiver operating curve across three imaging modalities for posterior fossa strokes.

|  | All lesions | Cerebellar lesions | Cerebellar lesions>5mLs |
| --- | --- | --- | --- |
| NCCT | R1: 0.56 (0.51-0.61)  R2:0.54 (0.50-0.58) | R1: 0.57 (0.50-0.63)  R2: 0.55 (0.50-0.60) | R1: 0.67 (0.53-0.81)  R2: 0.63 (0.50-0.75) |
| Automated core-penumbra map | R1:0.63 (0.56-0.70)  R2: 0.62 (0.54-0.70) | R1: 0.63 (0.54-0.72)  R2: 0.63 (0.53-0.73) | R1: 0.80 (0.65-0.94)  R2: 0.77 (0.62-0.91) |
| Automated core-penumbra + unprocessed maps | R1: 0.72 (0.62-0.81)  R2: 0.65 (0.57-0.73) | R1: 0.70 (0.59-0.80)  R2: 0.62 (0.52-0.73) | R1: 0.87 (0.78-0.97)  R2: 0.76 (0.61-0.91) |

*Table 3.* Area under the curve (with 95% confidence intervals) for the detection of posterior fossa strokes for different imaging modalities excluding patients treated with reperfusion therapy.

|  | Posterior fossa strokes | Cerebellar strokes | Cerebellar strokes>5mL |
| --- | --- | --- | --- |
| Brain non-contrast CT | 0.57 (0.53-0.61) | 0.59 (0.53-0.66) | 0.75 (0.60-0.90) |
| Automated core-penumbra map | 0.60 (0.54-0.66) | 0.62 (0.53-0.71) | 0.78 (0.64-0.92) |
| Automated core-penumbra + unprocessed maps | 0.68 (0.60-0.75) | 0.68 (0.59-0.78) | 0.88 (0.79-0.97) |

*Figure 1:* The area under the receiver operating curve across three imaging modalities for posterior fossa lesions


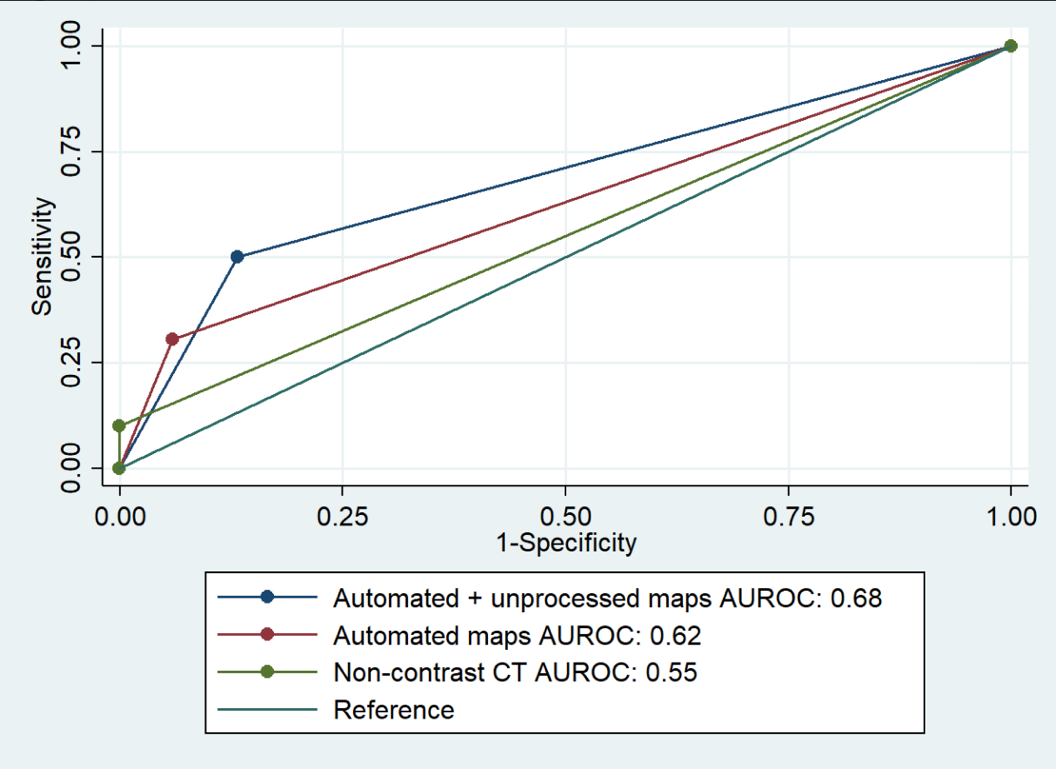


*Figure 2:* The area under the receiver operating curve across three imaging modalities for cerebellar lesions


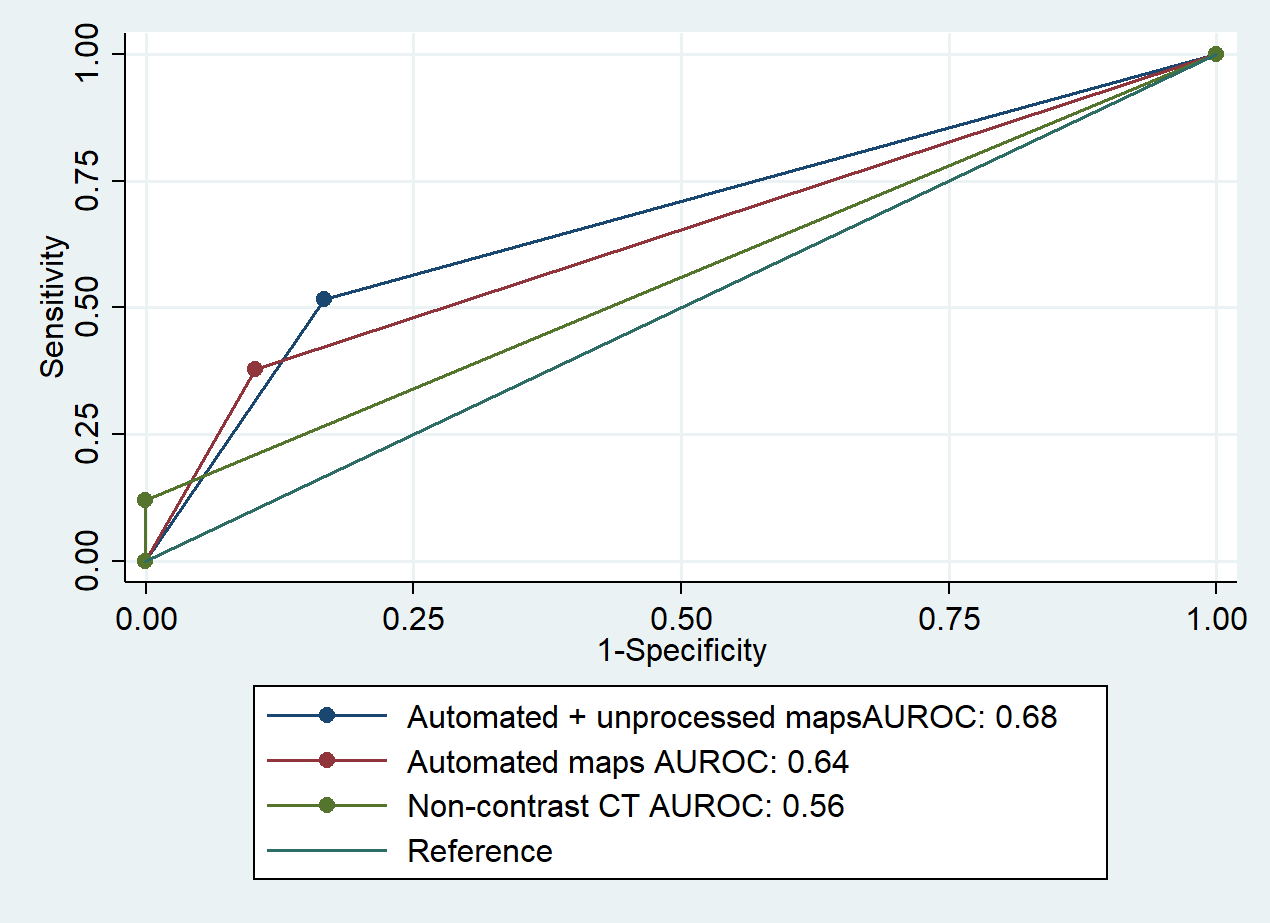


*Figure 3:* The area under the receiver operating curve across three imaging modalities for cerebellar lesions>5ml


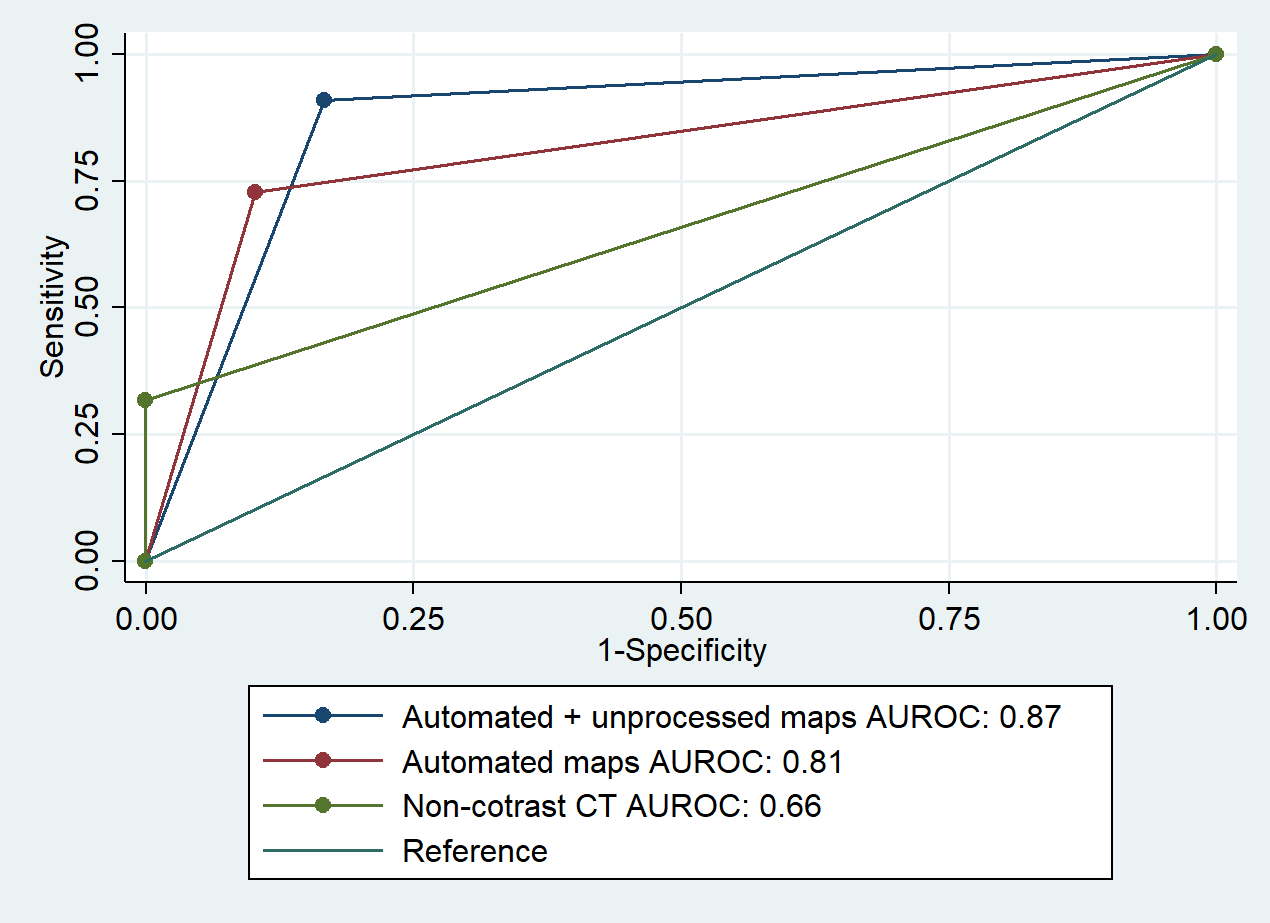

Supplement: Supplementary file 1 [file Data_Sheet_1.docx]
